# Importance of Correlation between Gene Expression Levels: Application to the Type I Interferon Signature in Rheumatoid Arthritis

**DOI:** 10.1371/journal.pone.0024828

**Published:** 2011-10-17

**Authors:** Frédéric Reynier, Fabien Petit, Malick Paye, Fanny Turrel-Davin, Pierre-Emmanuel Imbert, Arnaud Hot, Bruno Mougin, Pierre Miossec

**Affiliations:** 1 Joint Unit Hospices Civils de Lyon - bioMérieux, Hôpital Edouard Herriot, Lyon, France; 2 Department of Clinical Immunology and Rheumatology, and immunogenomics and Inflammation Research Unit EA 4130, University of Lyon, Hôpital Edouard Herriot, Lyon, France; Institut Jacques Monod, France

## Abstract

**Background:**

The analysis of gene expression data shows that many genes display similarity in their expression profiles suggesting some co-regulation. Here, we investigated the co-expression patterns in gene expression data and proposed a correlation-based research method to stratify individuals.

**Methodology/Principal Findings:**

Using blood from rheumatoid arthritis (RA) patients, we investigated the gene expression profiles from whole blood using Affymetrix microarray technology. Co-expressed genes were analyzed by a biclustering method, followed by gene ontology analysis of the relevant biclusters. Taking the type I interferon (IFN) pathway as an example, a classification algorithm was developed from the 102 RA patients and extended to 10 systemic lupus erythematosus (SLE) patients and 100 healthy volunteers to further characterize individuals. We developed a correlation-based algorithm referred to as Classification Algorithm Based on a Biological Signature (CABS), an alternative to other approaches focused specifically on the expression levels. This algorithm applied to the expression of 35 IFN-related genes showed that the IFN signature presented a heterogeneous expression between RA, SLE and healthy controls which could reflect the level of global IFN signature activation. Moreover, the monitoring of the IFN-related genes during the anti-TNF treatment identified changes in type I IFN gene activity induced in RA patients.

**Conclusions:**

In conclusion, we have proposed an original method to analyze genes sharing an expression pattern and a biological function showing that the activation levels of a biological signature could be characterized by its overall state of correlation.

## Introduction

A wide range of methods for microarray data analysis have evolved, ranging from simple fold-change approaches to many complex and computationally demanding techniques [1]. Gene expression profiling by microarray technology has become a widely used strategy for investigating the molecular mechanisms underlying many complex diseases [2]. However, the analysis is further complicated by the biological heterogeneity encountered in most of the diseases.

A common observation in the analysis of gene expression is that many genes show similar expression patterns [3] which may share biological functions under common regulatory control. Moreover, these co-expressed genes are frequently clustered according to their expression patterns in subset of experimental conditions [4]. Thus, gene co-expression instead of differential expression could be informative as well. Bi-clustering methods seek gene similarity in subsets of available conditions, which is more appropriate for functionally heterogeneous data [5], [6].

We have further explored this approach to study the heterogeneity of rheumatoid arthritis (RA) patients regarding their mRNA profiles in whole blood samples. In the context of RA, the clinical presentation of patients shows a high degree of heterogeneity, ranging from mild cases with a benign course to severe and erosive disease. In RA, gene expression profiling has been used to stratify patients based on molecular criteria using synovial tissue [7], [8] and more recently from peripheral blood cells [9].

Here, we took the signature of interferon (IFN)-related genes as an example to study correlation levels between genes composing that signature. A biclustering algorithm was applied to study a large gene expression dataset from peripheral whole blood of 102 RA patients. A correlation-based search algorithm referred to as Classification Algorithm Based on a Biological Signature (CABS) was developed to characterize patients based on their IFN signature. In RA patients with an activated IFN signature, gene expression levels were highly correlated and this was linked to the level of global IFN signature activation.

## Results

### Analysis of heterogeneity in RA with the biclustering method

Based on 102 RA patients, the study of biological data heterogeneity was conducted with a biclustering approach. This method using the SAMBA algorithm performs clustering on genes and conditions simultaneously in order to identify subsets of genes that show similar expression patterns across specific subsets of patients and vice versa. After data filtering, 121 biclusters were identified from 9,856 selected probe sets. To draw a clear picture of these co-expressed gene groups, the TANGO algorithm was used for GO functional enrichment analysis. The details of the results are given in table S1. Among them, these results have highlighted the importance of immune regulation across the “immune response” and “response to virus” ontology groups (biclusters 4, 21, 34, 35 and 39; see Table S1 as supplement information). Subsequently, we focused on bicluster 4 which represents the largest number of genes in these two GO categories.

### Ingenuity pathway analysis of IFN signature

**Figure 1:**
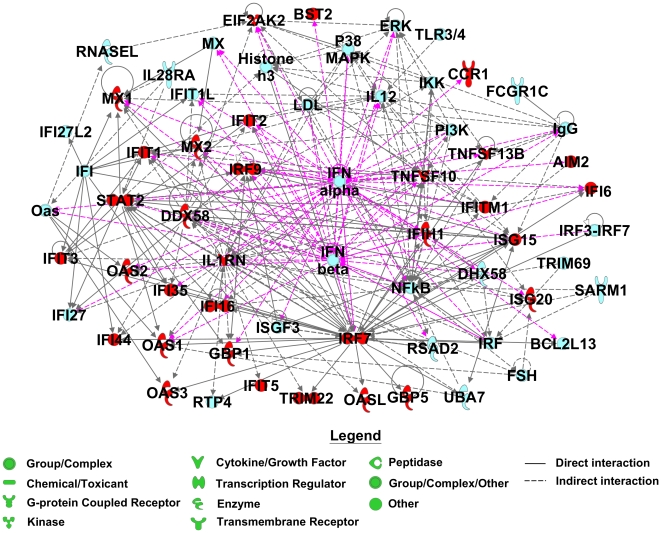
The network derived from the 35 genes which composed the IFN signature using Ingenuity Pathway Analysis (IPA) software. Edges (gene relationships) are displayed with labels that describe the nature of the relationship between nodes (genes). Nodes are displayed using various shapes that represent the functional class of the gene product. Genes in red belong to the list of the 35 IFN-related genes. Genes in blue were integrated into the computationally generated networks on the basis of the evidence stored in the IPA knowledge memory indicating a relevance to this network. The network showed central connection represented by the type I interferon. The pink arrows represent the direct and indirect interactions for genes of type I family of interferons (*IFN-α*, *IFN-β*).

**Figure 2:**
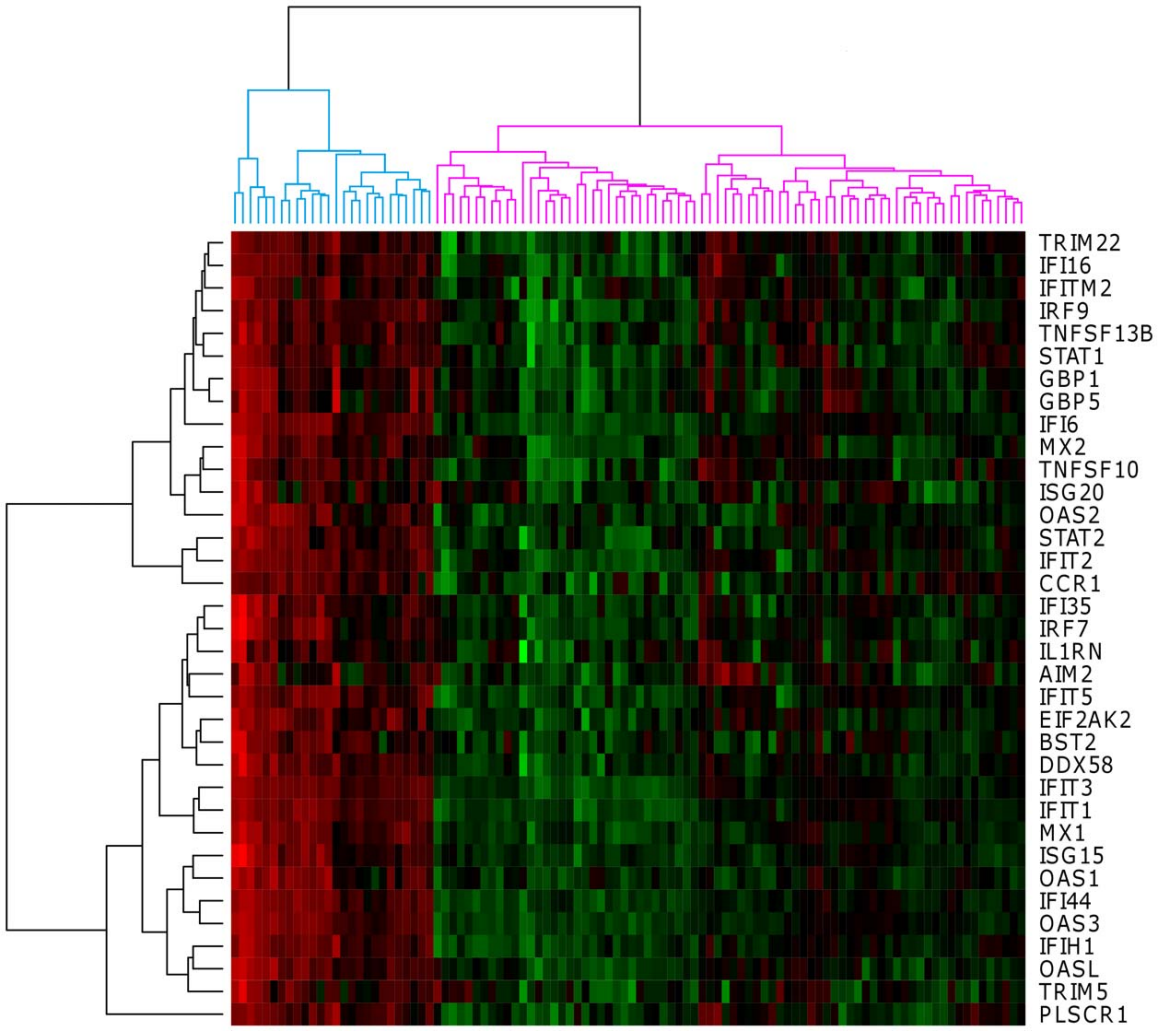
Gene expression profiles from the IFN signature. Unsupervised hierarchical clustering of 35 IFN-related genes that distinguish rheumatoid arthritis (RA) patients IFN^high^ (blue dendrogram) from RA patients IFN^low^ (purple dendrogram). Each row represents a gene; each column shows the expression for 35 IFN-related genes expressed by each patients. Red indicates genes that are expressed at higher levels and green indicates genes that are expressed at lower levels.

To further elucidate the importance of immune regulation, we conducted pathway analyses on bicluster 4 (n = 37 genes). To summarize, a pathway corresponding to interferon signaling (*IFI35*, *IFIT1*, *IFIT3*, *IFITM1*, *IRF9*, *MX1*, *OAS1*, *STAT2*) was prominently represented (B-H p-value = 1.86E-13). Moreover, a literature review showed that 35 genes among the 37 appeared directly or indirectly related to interferon. Thereafter, IPA was conducted on the 35 genes which composed the IFN signature. IPA can not only build associations of genes identified in our analysis (“focus” genes), but also predict the involvement of additional molecules not associated in the main gene list. Out of the list, 32 genes were found in the IPA knowledge database, and are labelled “focus genes”. Based on these focus genes, IPA generated a biological network (score 85, focus genes 32) providing evidence that type I IFN represented by the *IFNα* and *IFNβ* genes is responsible for the activation of IFN-related genes (Figure 1). The list of these 35 genes is presented in the right column of figure 2.

### Activation of IFN pathway in a sub-group of RA patients

To visualize the expression profiles of the 35 IFN-response genes among all RA patients and to investigate their interactions, a hierarchical clustering was performed with the Spotfire Decision Site 8.2.1. This clustering separated the samples into two main groups, one of patients with RA (n = 26/102, 25.5%) with high expression (Figure 2, blue dendrogram) of this set of IFN-related genes (IFN^high^) and another (n = 76/102, 74.5%) with lower expression (Figure 2, purple dendrogram) (IFN^low^).

### Characterization of the IFN signature based on a correlation approach

**Figure 3:**
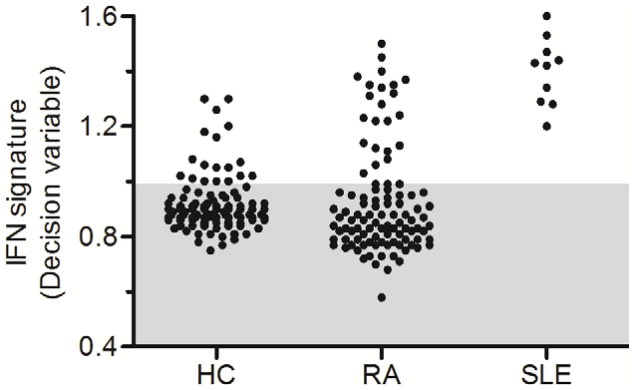
Stratification of individuals according to the IFN signature. Each point represents a single individual with the decision variable calculated from the Classification Algorithm based on a Biological Signature (CABS). The shaded box indicates the normal range according to the rule of the CABS: If *D_high_low_≥1*, the signature is defined as “high signature” and If *D_high_low_<1*, the signature is defined as “low signature” knowing that *D_high_low_ = COR_high_/COR_low_*.

**Figure 4:**
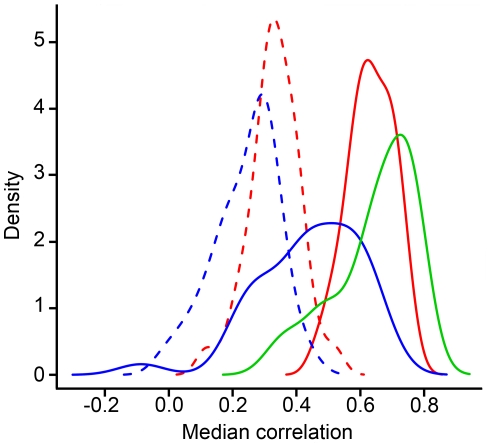
Correlation profiles from the different groups. A correlation index was defined for each gene of the IFN signature as the median of its correlations with the remaining genes. Thus, the correlation profiles for the different groups: healthy controls (HC) IFN^low^ (blue dotted), HC IFN^high^ (blue line), rheumatoid arthritis patients (RA) IFN^low^ (red dotted) and RA IFN^high^ (red line) and systemic lupus erythematosus patients (SLE) IFN^high^ (green line), are represented using the 35 calculated correlation indexes from the IFN signature genes. The median values of the correlation indexes obtained for the different groups are 0.27, 0.44, 0.33, 0.63 and 0.68, respectively.

The expression pattern of 35 IFN-response genes was defined as the “IFN signature”. To go further in the description of the IFN-related genes, the correlation levels between the co-expressed genes were assessed in the two groups of RA patients. Interestingly, the analysis revealed disparities between correlation levels. The group associated with high IFN expression level showed a better correlation (R_median_ = 0.63) than the other one (R_median_ = 0.33), with a significant difference (p = 8.46E-13), suggesting a functional difference in the activated state of these genes. A classification algorithm was applied to obtain a better characterization of the IFN signature based on the correlation of the 35 gene expression levels. The results showed that the IFN signature presented a large variation between individuals (Figure 3). 15/100 HC (15%), 22/102 RA patients (22%) and 10/10 SLE patients (100%) with a decision variable ≥1 for the high signature (IFN^high^) were identified, while the remainder of individuals, with a decision variable <1, were defined as IFN^low^. From the sub-groups identified by the CABS, the comparison of the correlation profiles showed heterogeneous distributions (Figure 4). Two groups were observed, first with RA and SLE patients with a high IFN signature and a median correlation of 0.63 and 0.68 respectively; second with RA patients and HC IFN^low^ and a median correlation of 0.33 and 0.27 respectively. However, the shape of the curve for the HC IFN^high^ (R_median_ = 0.44 ; Figure 4, blue line) is very different from that seen for the IFN high RA or SLE patients and for the IFN low RA or controls. This suggests a very heterogeneous activation status of genes in this group of controls.

### Comparison of characterization methods of IFN signature

**Figure 5:**
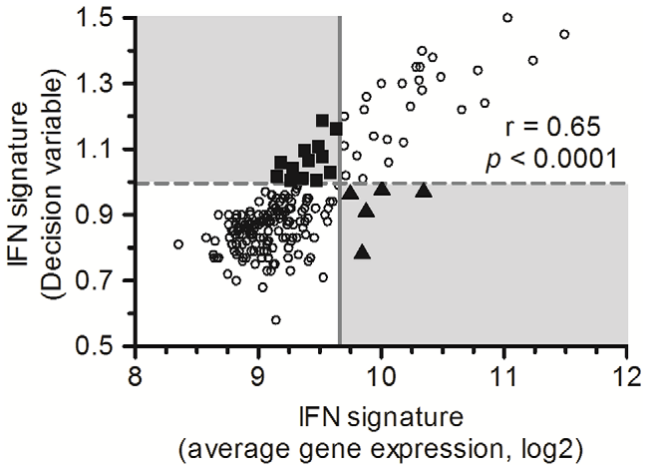
Comparative analysis of characterization methods of IFN signature. Each dot represents a single individual. The y-axis represents the decision variable of the IFN signature calculated from CABS. The grey dotted line indicates the threshold according to the rule of the CABS: If D_high_low_≥1, the signature is defined as “high signature” and If D_high_low_<1, the signature is defined as “low signature” knowing that D_high_low_ = COR_high_/COR_low_. The x-axis represents the average values of gene expression of the IFN signature. The solid grey line indicates the threshold of IFN response, by calculating the 95% limits of the HC (normal values, defined as the mean (SD) expression of the 35 IFN-related genes, ±1.96 SD). If the average gene expression is ≥9.68, the signature is defined as “high signature” and if average gene expression ratio is <9.68, the signature is defined as “low signature”. The shaded boxes show the divergence observed between both methods. The black triangles represent individuals with high average values of gene expression and low decision variable. The black squares represent individuals with low average values of gene expression and high decision variable.

A comparative analysis between correlation-based approach (CABS) and the classical “IFN score” based on the average values of gene expression was performed (Figure 5). First, this figure showed a correlation between the decision variable (correlation value) and the average values of gene expression (Spearman correlation test, r = 0.65, p-value<0.0001). Second, based on the respective thresholds, this comparison revealed differences between both approaches (9%). Individuals (black triangles) with a high average expression value of IFN-related genes were associated with a low level of correlation and vice versa with individuals represented by a black square.

### Effect of TNF inhibition on IFN pathway activation

**Figure 6:**
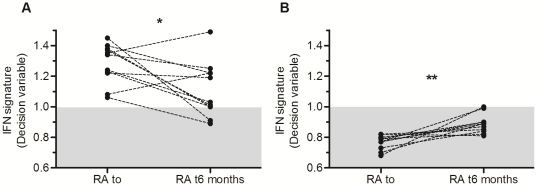
Follow-up the IFN signature in patients with rheumatoid arthritis (RA) treated with anti-TNF. Each point represents a single individual with the decision variable calculated from the Classification Algorithm based on a Biological Signature (CABS). The shaded box indicates the normal range according to the rule of the CABS: If *D_high_low_≥1*, the signature is defined as “high s*ignature*” and If *D_high_low_<1*, the signature is defined as “low signature” knowing that *D_high_low_ = COR_high_/COR_low_*. The Wilcoxon signed rank test was used to evaluate the statistical significance between patients before and after anti-TNF treatment **A**) (*p = 0.0186) **B**) (**p = 0.002).

The functional relationship between TNF inhibition and possible changes in IFN pathway activation was studied. CABS was used to assess the correlation levels in RA patients before and after anti-TNFα treatment. Out of the subgroup of 43 RA patients treated with anti-TNF, 22 RA patients (11 RA IFN^high^ and 11 RA IFN^low^; infliximab n = 6, etanercept n = 10 and adalimumab n = 6) were evaluated at 6 months for treatment response using the DAS28 criteria. Although the values appeared quite heterogeneous, a statistical significant decrease (p = 0.0186) of the correlation level was observed in patients associated with high IFN signature (Figure 6A). In contrast, a statistical significant increase (p = 0.002) of correlation levels was seen in RA patients with low IFN signature before treatment (Figure 6B). Despite a significant increase, the majority of these RA patients IFN^low^ did not reach the threshold of positivity. No statistical association was observed between the molecular stratification of RA patients (IFN^high^/IFN^low^) and the clinical characteristics presented in table 1 or the response to treatment at 6 months.

**Table 1:** Demographic and clinical characteristics of the patients and control subjects.

	RA(n = 102)	SLE(n = 10)	C(n = 100)
*Demographic data*			
Age^a^	50 (40,3–60)	37 (34–44)	57 (52–63)
Sex: Female, Male	79F, 23M	10F	86F, 14M
*Disease characteristics*			
ESR^a^	18 (8–44)	NA	NA
Rheumatoid Factor pos.(%)	70 (68,6)	NA	NA
Disease duration (years)^a^	5 (2–9)	4 (3–6)	NA
Disease activity	4,2 (3,3–5,2)^b^	13 (12–17.5)^c^	NA
*Medication*			
MTX (%)	87 (85,3)	NA	NA
MTX dose^a^	15 (15–20)	NA	NA

a Median (Q1–Q3).

b DAS28: Disease Activity Score.

c SLEDAI: Systemic Lupus Erythematosus Disease Activity Index.

ESR: Erythrocyte Sedimentation Rate; MTX: Methotrexate.

## Discussion

In this study, the heterogeneous nature of RA was addressed at a molecular level and the data showed that disease characteristics could be reflected by gene expression levels in whole blood. Using microarray technology, RA patients could be categorized into 121 biclusters, sub-groups of patients sharing a same profile for a group of genes. With the type I IFN signature as an example, we showed a variation of the correlation level within 102 RA patients representative to the RA population. Each patient can be characterized by a single correlation value of the expression observed for the 35 IFN-related genes. Interestingly, our results revealed a heterogeneous IFN expression (Figure 2) characterized by a correlation level of the gene expression which may reflect the global IFN signature activation. This method allowed us to define two well separated groups (IFN^low^ vs. IFN^high^; p = 8.46E-13) based on the correlation levels with the IFN^high^ corresponding to 22% of our RA patients cohort. In fact, it was shown that genes with similar functions usually are co-expressed under certain experimental conditions only [4]. The sample profiles can resemble to the physiological relationships expected between the studied samples [10]. Prieto C. et al. demonstrated that studies of heterogeneous datasets, mixing many case samples from pathological or altered states with “normal” samples disturb gene co-expression analysis. In the context of these observations, our results suggest that the co-expressed gene clusters, defining functional groups, depend on the activation status.

The method commonly used in the literature does not take into account the activation status of the biological signature, which could generate some misclassification. Indeed, the increase of IFN regulated genes has been reported in different diseases like SLE [11], systemic sclerosis [12], multiple sclerosis [13] and in tissues from patients with Sjögren's syndrome [14], type I diabetes [15], [16] and dermatomyositis [17], [18]. To characterize the IFN signature, an IFN “score” is calculated for each patient and control based on the average expression of genes which composed the signature [9], [11], [15], [18], [19], [20]. However, this approach does not take into account the co-regulation of these IFN-related genes. When genes are co-regulated under various biological conditions, the corresponding expression profiles may display relative similarity or co-expression [21]. Our method offers an alternative with which the IFN signature could be characterized by the level of global correlation (Figures 3 and 4) and not solely by the expression levels. In fact, analyses of our results based on the mean expression of the IFN-related genes showed disparities in the classification of HC and RA patients (9%, Figure 5). These differences between gene expression and correlation levels in the IFN signature could be explained by different factors. Studies showed that IFN-related genes could be regulated by several independent pathways on IFN signaling [22], [23]. Their expression could be also controlled by the polymorphic sequences which mainly composed the promoter regions of theses genes [24], [25]. These different factors could explain the presence of individual heterogeneity in the expression of these genes and thus the discrepancies observed between the two approaches.

To better understand differences between disease and healthy status, different approaches like transcriptomics or proteomics analyses allow the study of molecular networks and signaling pathways, with the major challenge of integrating this information into a systems approach [15]. Our method permits to identify truly active biological networks associating only with high levels of correlation of biological signature components. Indeed, taking into account this new correlation aspect for the interpretation of biological networks should allow capturing the actually activated mechanisms at the cellular level.

Interestingly, such correlation-based approach can be advantageously applied to investigate the dynamics of evolution of cellular mechanisms like response to treatment. As an example, in the context of RA, we have applied this method to monitor patients treated by anti-TNF therapy. Although the cross-regulation of *TNFα* and *IFNα* has been previously described [26], the effects of anti-TNF treatment on the expression of IFN-related genes had never been shown by such approach. The results showed that a high IFN signature was conserved after anti-TNF treatment (Figure 6A), while a significant increase was observed in RA IFN^low^ six months after treatment (Figure 6B). However, the level of positivity has never reached the one observed in SLE patients, known to strongly express the IFN signature. This observation could explain that RA patients treated with anti-TNF develop rather benign clinical symptoms of SLE that are reversible after discontinuation of therapy [27], [28]. Contrary to a recent publication [29], we did not find clinical relevance associated to this IFN signature. The authors showed that an increased IFN-response gene activity after anti-TNF treatment was linked to a poor clinical outcome. In our results, only a trend was observed according to the delta DAS28 score (p = 0.07, data not shown). Besides the difference in method used or the sample size which may explain the discrepancies, our study presented RA patients with a large panel of anti-TNF treatments (infliximab, etanercept and adalimumab). Indeed, several studies suggest differential effects of anti-TNF treatments on IFN-response activity which could explain the lack of specificity in our study [29].

Interestingly, our method using CABS allowed us to pinpoint type I IFN signaling as a means to stratify RA patients even starting with whole blood transcriptomics analysis from samples collected in PAXgene tubes. Similar analyses can be performed for the other identified biclusters, highlighting the obvious advantage of whole blood transcriptomics. Using the example of the IFN signature, the use of correlations showed interest in the characterization of the genes sharing both an expression pattern and a biological function. The use of expression correlations may be a better way to obtain a global picture of an activated signature in various disease conditions.

## Methods

### Ethics statement

All subjects provided written informed consent and the study was approved by the local Ethical Committee for clinical research of the University hospitals of Lyon.

### Patients and controls

102 RA patients fulfilling the revised American College of Rheumatology 1987 criteria for RA [30] were enrolled. Their clinical characteristics are shown in table 1. Among the 102 RA patients, a subgroup of RA patients treated for 6 months with anti-TNF, 22 RA patients characterized as IFN^high^ (n = 11) and IFN^low^ (n = 11), were included (IFN^high^ group: infliximab n = 4, etanercept n = 3 and adalimumab n = 4; IFN^low^ group: infliximab n = 2, etanercept n = 7 and adalimumab n = 2). As an IFN positive control group (IFN^high^), 10 systemic lupus erythematosus patients (SLE) fulfilling the American College of Rheumatology criteria for the SLE [31] were studied. In addition, 100 age- and sex-matched healthy control subjects (HC) without any familial history of RA, autoimmune disease and concomitant medication were also recruited.

### Sample collection, processing and microarray hybridization

Peripheral blood samples were collected in PAXgene™ Blood RNA tubes (PreAnalytix, Hilden, Germany) in order to stabilize mRNA [32]. Blood samples were incubated at room temperature for 2 h, and then stored at −20°C until RNA extraction according to the manufacturer's instructions. Briefly, RNA was isolated using the PAXgene™ Blood RNA kit (PreAnalytix). Following cell lysis, nucleic acids were pelleted and treated with a buffer containing proteinase K. After digestion with a RNase-free DNase (Qiagen, Valencia, CA, USA), RNA was subsequently purified on PAXgene™ spin columns and eluted in 80 µl of elution buffer. The quality of RNA was determined with the Bioanalyzer® 2100 (Agilent Technologies, Waldbronn, Germany), following the manufacturer's protocol. cDNA was synthesized from 50 ng of total RNA using the WT-Ovation™ System (NuGEN, San Carlos, CA, USA) powered by Ribo-SPIA™ technology. Fragmented cDNA was end labeled with a biotin-conjugated nucleotide analog (DLR-1a; Affymetrix, Santa Clara, CA, USA) using terminal transferase (Roche Diagnostics, Mannheim, Germany). Fragmented and labeled cDNA was hybridized for 18 h at 50°C in a hybridization solution containing 7% DMSO. Hybridization was performed using GeneChip® Human Genome U133 Plus 2.0 arrays (Affymetrix), containing 54,675 probe sets corresponding to 38,500 identified genes. After washing, chips were stained with streptavidin-phycoerythrin according to Affymetrix EukGE-WS2v4 protocol using the Fluidic FS450 station. The microarrays were read with the GeneChip® Scanner 3000 (Affymetrix). Affymetrix GeneChip Operating Software version 1.4 (GCOS) was used to manage Affymetrix GeneChip array data and to automate the control of GeneChip fluidics stations and scanners.

### Data analysis

#### Data processing

Expression data were generated using the Robust Multi-array Average (RMA) method [33] implemented in the Affy package of the Bioconductor microarray analysis environment (http://www.bioconductor.org). The RMA method consists of three steps: background adjustment, quantile normalization [34] and probe set summary of the log-normalized data applying a median polishing procedure. Before the analysis of heterogeneity, two filters were applied based on expression level and variability to lower the dimensionality of the data and to avoid false discoveries. First, genes with a median expression value below a given threshold were eliminated. This threshold was set to 6 in log base 2 corresponding to twice the average background level. The second filter eliminated genes with a low variation. Thus, the Median Absolute Deviation (MAD) for the remaining genes was calculated and those with a MAD lower than the median of the MAD calculated over the remaining genes after intensity based filtering were eliminated.

#### Biclustering and functional enrichment analyses

The SAMBA algorithm (Statistical-Algorithmic Method for Bicluster Analysis) implemented in EXPANDER 4.0.3 (EXPression ANalyzer and DisplayER) was used for the biclustering [35]. This algorithm uses probabilistic modeling of the data and theoretical graph techniques to identify such subsets of genes that behave similarly across a subset of patients [36].

The TANGO algorithm (Tool for Analysis of GO enrichment), implemented in EXPANDER 4.0.3, was used to identify the biological significance of these biclusters [35].

#### Interferon molecular pathway analysis

Canonical pathway analyses was performed to define overrepresentation of canonical pathways of the selected genes. Canonical pathway analyses of specific genes coming from statistical analysis were performed using Ingenuity Pathway Analysis (IPA), (www.ingenuity.com). B-H multiple testing correction p-value test was used to calculate the p-value for determining the probability that each canonical pathway assigned to the dataset was due to chance alone. P-value<0.01 was applied in calculations and the Human Genome U133 Plus 2.0 array was used as the reference when ranking the statistical significance of canonical pathways.

Networks of the IFN genes were constructed using Ingenuity Pathway Analysis (IPA), (www.ingenuity.com). Genes were found in the IPA knowledge database are labeled “focus” genes. Based on the focus genes, IPA generated a set of molecular networks with a cutoff of 70 genes for each network based on interactions between uploaded genes and all other genes/proteins stored in the knowledge base. Each network is assigned a score according to the number of focus genes in our dataset. These scores are derived from negative logarithm of the *P* and are indicators of the degree of significance. Scores of 4 or higher have 99.9% confidence level of significance as defined in detail elsewhere [37].

#### Classification Algorithm based on a Biological Signature (CABS)

Taking the example of the IFN-related genes, a classification algorithm was developed to identify individuals with or without this biological signature. Applied to the IFN-related genes, the CABS is divided into three steps.

*Step 1 Prototype construction:* Two groups of RA patients (IFN^high^; IFN^low^) were identified from the hierarchical clustering representing the 35 IFN-related genes which characterized the IFN signature (Figure 2). The prototype was defined from these two groups. Median expression values was calculated in the two groups. Prototype *Pi* was defined from group *i*; the vector (*Gi_1_,…,Gi_M_*) represents the expression of the prototype *Pi*, where *i* is high or low, *Gij* is the median expression of gene *j* in group *i*, *M* is the size of the IFN signature.

*Step 2 Decision Variable Calculation*: Given the definition of the prototypes described above, a criteria was needed to assess the similarity of a given individual to those prototypes. For a given individual, the IFN signature profile was defined as the vector corresponding to the expression level of the 35 genes constituting the signature. The similarity of this profile with both prototypes was calculated using the Pearson correlation coefficient and noted *COR_high_* et *COR_low_*. The decision variable calculation was given by the ratio between these two correlations: *D_high_low_ = COR_high_/COR_low_* indicating proximity to one or other of the prototypes.

*Step 3 : Decision Making*: Given the decision variable describe above, an individual was assigned High IFN if the ratio *D_high_low_*≥*1* meaning that *COR_high_*≥*COR_low_*. Inversely, an individual was assigned low IFN if the ratio *D_high_low_*<*1 meaning that COR_high_*<*COR_low_*.

## Supporting Information

Table S1 **Ontological analysis of the 121 biclusters obtained from the 102 RA patients.** The TANGO algorithm (Tool for Analysis of GO enrichment) was used to identify the biological significance of 121 biclusters from 9,856 selected probe sets (see material and methods for details). Among them, these results have highlighted the importance of immune regulation across the “immune response” and “response to virus” ontology groups (biclusters 4, 21, 34, 35 and 39. Processes with corrected p value<0.05 were considered significant [36].
(DOC)

## References

[1] Kerr MK (2003). Design considerations for efficient and effective microarray studies.. Biometrics.

[2] Wheelan SJ, Martinez Murillo F, Boeke JD (2008). The incredible shrinking world of DNA microarrays.. Mol Biosyst.

[3] Eisen MB, Spellman PT, Brown PO, Botstein D (1998). Cluster analysis and display of genome-wide expression patterns.. Proc Natl Acad Sci U S A.

[4] Ben-Dor A, Chor B, Karp R, Yakhini Z (2003). Discovering local structure in gene expression data: the order-preserving submatrix problem.. J Comput Biol.

[5] Cheng Y, Church GM (2000). Biclustering of expression data.. Proc Int Conf Intell Syst Mol Biol.

[6] Madeira SC, Oliveira AL (2004). Biclustering algorithms for biological data analysis: a survey.. IEEE/ACM Trans Comput Biol Bioinform.

[7] van der Pouw Kraan TC, van Gaalen FA, Huizinga TW, Pieterman E, Breedveld FC (2003). Discovery of distinctive gene expression profiles in rheumatoid synovium using cDNA microarray technology: evidence for the existence of multiple pathways of tissue destruction and repair.. Genes Immun.

[8] van der Pouw Kraan TC, van Gaalen FA, Kasperkovitz PV, Verbeet NL, Smeets TJ (2003). Rheumatoid arthritis is a heterogeneous disease: evidence for differences in the activation of the STAT-1 pathway between rheumatoid tissues.. Arthritis Rheum.

[9] van der Pouw Kraan TC, Wijbrandts CA, van Baarsen LG, Voskuyl AE, Rustenburg F (2007). Rheumatoid arthritis subtypes identified by genomic profiling of peripheral blood cells: assignment of a type I interferon signature in a subpopulation of patients.. Ann Rheum Dis.

[10] Prieto C, Risueño A, Fontanillo C, De las Rivas J (2008). Human gene coexpression landscape: confident network derived from tissue transcriptomic profiles.. PLoS One.

[11] Baechler EC, Batliwalla FM, Karypis G, Gaffney PM, Ortmann WA (2003). Interferon-inducible gene expression signature in peripheral blood cells of patients with severe lupus.. Proc Natl Acad Sci U S A.

[12] Tan FK, Zhou X, Mayes MD, Gourh P, Guo X (2006). Signatures of differentially regulated interferon gene expression and vasculotrophism in the peripheral blood cells of systemic sclerosis patients.. Rheumatology (Oxford).

[13] van Baarsen LG, van der Pouw Kraan TC, Kragt JJ, Baggen JM, Rustenburg F (2006). A subtype of multiple sclerosis defined by an activated immune defense program.. Genes Immun.

[14] Bave U, Nordmark G, Lövgren T, Rönnelid J, Cajander S (2005). Activation of the type I interferon system in primary Sjögren's syndrome: a possible etiopathogenic mechanism.. Arthritis Rheum.

[15] Reynier F, Pachot A, Paye M, Xu Q, Turrel-Davin F (2010). Specific gene expression signature associated with development of autoimmune type-I diabetes using whole-blood microarray analysis.. Genes Immun.

[16] Huang X, Yuang J, Goddard A, Foulis A, James RF (1995). Interferon expression in the pancreases of patients with type I diabetes.. Diabetes.

[17] Greenberg SA, Pinkus JL, Pinkus GS, Burleson T, Sanoudou D (2005). Interferon-alpha/beta-mediated innate immune mechanisms in dermatomyositis.. Ann Neurol.

[18] Baechler EC, Bauer JW, Slattery CA, Ortmann WA, Espe KJ (2007). An interferon signature in the peripheral blood of dermatomyositis patients is associated with disease activity.. Mol Med.

[19] Kirou KA, Lee C, George S, Louca K, Papagiannis IG (2004). Coordinate overexpression of interferon-alpha-induced genes in systemic lupus erythematosus.. Arthritis Rheum.

[20] Bauer JW, Baechler EC, Petri M, Batliwalla FM, Crawford D (2006). Elevated serum levels of interferon-regulated chemokines are biomarkers for active human systemic lupus erythematosus.. PLoS Med.

[21] Chou JW, Zhou T, Kaufmann WK, Paules RS, Bushel PR (2007). Extracting gene expression patterns and identifying co-expressed genes from microarray data reveals biologically responsive processes.. BMC Bioinformatics.

[22] Ning S, Huye LE, Pagano JS (2005). Regulation of the transcriptional activity of the IRF7 promoter by a pathway independent of interferon signaling.. J Biol Chem.

[23] Gugliesi F, Mondini M, Ravera R, Robotti A, de Andrea M (2005). Up-regulation of the interferon-inducible IFI16 gene by oxidative stress triggers p53 transcriptional activity in endothelial cells.. J Leukoc Biol.

[24] Mälarstig A, Sigurdsson S, Eriksson P, Paulsson-Berne G, Hedin U (2008). Variants of the interferon regulatory factor 5 gene regulate expression of IRF5 mRNA in atherosclerotic tissue but are not associated with myocardial infarction.. Arterioscler Thromb Vasc Biol.

[25] Akahoshi M, Nakashima H, Sadanaga A, Miyake K, Obara K (2008). Promoter polymorphisms in the IRF3 gene confer protection against systemic lupus erythematosus.. Lupus.

[26] Palucka AK, Blanck JP, Bennett L, Pascual V, Banchereau J (2005). Cross-regulation of TNF and IFN-alpha in autoimmune diseases.. Proc Natl Acad Sci U S A.

[27] Pisetsky DS (2000). Tumor necrosis factor alpha blockers and the induction of anti-DNA autoantibodies.. Arthritis Rheum.

[28] Shakoor N, Michalska M, Harris CA, Block JA (2002). Drug-induced systemic lupus erythematosus associated with etanercept therapy.. Lancet.

[29] van Baarsen LG, Wijbrandts CA, Rustenburg F, Cantaert T, van der Pouw Kraan TC (2010). Regulation of IFN response gene activity during infliximab treatment in rheumatoid arthritis is associated with clinical response to treatment.. Arthritis Res Ther.

[30] Arnett FC, Edworthy SM, Bloch DA, McShane DJ, Fries JF (1988). The American Rheumatism Association 1987 revised criteria for the classification of rheumatoid arthritis.. Arthritis Rheum.

[31] Tan E, Cohen A, Fries J, Masi A, McShane D (1982). The 1982 revised criteria for the classification of systemic lupus erythematosus.. Arthritis Rheum.

[32] Rainen L, Oelmueller U, Jurgensen S, Wyrich R, Ballas C (2002). Stabilization of mRNA expression in whole blood samples.. Clin Chem.

[33] Irizarry RA, Hobbs B, Collin F, Beazer-Barclay YD, Antonellis KJ (2003). Exploration, normalization, and summaries of high density oligonucleotide array probe level data.. Biostatistics.

[34] Bolstad BM, Irizarry RA, Astrand M, Speed TP (2003). A comparison of normalization methods for high density oligonucleotide array data based on variance and bias.. Bioinformatics.

[35] Shamir R, Maron-Katz A, Tanay A, Linhart C, Steinfeld I (2005). EXPANDER: an integrative program suite for microarray data analysis.. BMC Bioinformatics.

[36] Tanay A, Sharan R, Shamir R (2002). Discovering statistically significant biclusters in gene expression data.. Bioinformatics.

[37] Calvano SE, Xiao W, Richards DR, Felciano RM, Baker HV (2005). A network-based analysis of systemic inflammation in humans.. Nature.

