# Supplementary material for: Importance of Correlation between Gene Expression Levels: Application to the Type I Interferon Signature in Rheumatoid Arthritis
Source: PLoS One. 2011 Oct 17;6(10):e24828. doi: 10.1371/journal.pone.0024828 (PMC3197194; doi:10.1371/journal.pone.0024828)
Supplement: Table S1 — Ontological analysis of the 121 biclusters obtained from the 102 RA patients. The TANGO algorithm (Tool for Analysis of GO enrichment) was used to identify the biological significance of 121 biclusters from 9,856 selected probe sets (see material and methods for details). Among them, these results have highlighted the importance of immune regulation across the “immune response” and “response to virus” ontology groups (biclusters 4, 21, 34, 35 and 39. Processes with corrected p value<0.05 were considered significant [36]. (DOC) [file pone.0024828.s001.doc]

**TABLE S1**: Ontological analysis of the 121 biclusters obtained from the 102 RA patients.

| **Bicluster** | **Probesets** | **GO ID - term (recovered genes)** | **Corrected p-value** |
| --- | --- | --- | --- |
| 1 | 105 |  |  |
| 2 | 67 |  |  |
| 3 | 99 |  |  |
| 4 | 227 | GO:0006955 - immune response (32) | 0.001 |
|  |  | GO:0009615 - response to virus (17) | 0.001 |
| 5 | 254 |  |  |
| 6 | 93 |  |  |
| 7 | 136 | GO:0003735 - structural constituent of ribosome (21) | 0.001 |
|  |  | GO:0003723 - RNA binding (23) | 0.001 |
| 8 | 221 |  |  |
| 9 | 220 |  |  |
| 10 | 95 |  |  |
| 11 | 149 | GO:0004871 - signal transducer activity (31) | 0.001 |
| 12 | 141 |  |  |
| 13 | 171 |  |  |
| 14 | 52 |  |  |
| 15 | 70 |  |  |
| 16 | 108 | GO:0003735 - structural constituent of ribosome (9) | 0.01 |
| 17 | 33 |  |  |
| 18 | 51 |  |  |
| 19 | 208 |  |  |
| 20 | 50 |  |  |
| 21 | 65 | GO:0006955 - immune response (14) | 0.001 |
|  |  | GO:0009615 - response to virus (10) | 0.001 |
| 22 | 128 | GO:0006091 generation of precursor metabolites and energy (16) | 0.001 |
|  |  | GO:0006119 - oxidative phosphorylation (11) | 0.001 |
|  |  | GO:0016655 oxidoreductase activity\, acting on NADH or NADPH\, quinone or similar compound as acceptor (6) | 0.0028 |
|  |  | GO:0015078 - hydrogen ion transporter activity (10) | 0.001 |
| 23 | 122 | GO:0003735 - structural constituent of ribosome (11) | 0.001 |
|  |  | GO:0006119 - oxidative phosphorylation (8) | 0.001 |
|  |  | GO:0015078 - hydrogen ion transporter activity (7) | 0.002 |
| 24 | 76 | GO:0004871 - signal transducer activity (20) | 0.001 |
|  |  | GO:0006968 - cellular defense response (7) | 0.001 |
|  |  | GO:0007154 - cell communication (25) | 0.013 |
|  |  | GO:0019835 - cytolysis (4) | 0.001 |
| 25 | 30 | GO:0003735 - structural constituent of ribosome (6) | 0.001 |
| 26 | 86 |  |  |
| 27 | 210 |  |  |
| 28 | 60 |  |  |
| 29 | 72 |  |  |
| **Bicluster** | **Probesets** | **GO ID - term (recovered genes)** | **Corrected p-value** |
| 30 | 118 |  |  |
| 31 | 134 |  |  |
| 32 | 91 |  |  |
| 33 | 190 |  |  |
| 34 | 230 | GO:0006955 - immune response (30) | 0.001 |
|  |  | GO:0009615 - response to virus (17) | 0.001 |
| 35 | 157 | GO:0006955 - immune response (28) | 0.001 |
|  |  | GO:0009615 - response to virus (12) | 0.001 |
|  |  | GO:0009607 - response to biotic stimulus (15) | 0.001 |
| 36 | 84 |  |  |
| 37 | 211 |  |  |
| 38 | 52 |  |  |
| 39 | 253 | GO:0006955 - immune response (31) | 0.001 |
|  |  | GO:0009615 - response to virus (17) | 0.001 |
| 40 | 72 |  |  |
| 41 | 234 |  |  |
| 42 | 112 |  |  |
| 43 | 21 |  |  |
| 44 | 83 |  |  |
| 45 | 73 | GO:0007166 - cell surface receptor linked signal transduction (14) | 0.007 |
|  |  | GO:0006968 - cellular defense response (6) | 0.005 |
|  |  | GO:0019835 - cytolysis (4) | 0.001 |
|  |  | GO:0004872 - receptor activity (16) | 0.001 |
|  |  | GO:0007165 - signal transduction (23) | 0.013 |
| 46 | 38 |  |  |
| 47 | 59 |  |  |
| 48 | 115 |  |  |
| 49 | 97 |  |  |
| 50 | 127 |  |  |
| 51 | 193 |  |  |
| 52 | 89 |  |  |
| 53 | 48 |  |  |
| 54 | 36 |  |  |
| 55 | 109 | GO:0007165 - signal transduction (35) | 0.001 |
| 56 | 128 | GO:0007155 - cell adhesion (13) | 0.048 |
|  |  | GO:0009611 - response to wounding (15) | 0.001 |
| 57 | 73 |  |  |
| 58 | 94 | GO:0006091 - generation of precursor metabolites and energy (15) | 0.001 |
|  |  | GO:0006119 - oxidative phosphorylation (9) | 0.001 |
|  |  | GO:0015078 - hydrogen ion transporter activity (8) | 0.001 |
| 59 | 99 |  |  |
| 60 | 51 | GO:0004871 - signal transducer activity (14) | 0.037 |
|  |  | GO:0042113 - B cell activation (5) | 0.039 |
| 61 | 84 |  |  |
| 62 | 18 |  |  |
| 63 | 122 | GO:0009058 - biosynthetic process (21) | 0.014 |
|  |  | GO:0006091 - generation of precursor metabolites and energy (14) | 0.001 |
| **Bicluster** | **Probesets** | **GO ID - term (recovered genes)** | **Corrected p-value** |
|  |  | GO:0006119 - oxidative phosphorylation (9) | 0.001 |
|  |  | GO:0006119 - oxidative phosphorylation (9) | 0.001 |
|  |  | GO:0015078 - hydrogen ion transporter activity (9) | 0.001 |
| 64 | 95 |  |  |
| 65 | 58 |  |  |
| 66 | 33 |  |  |
| 67 | 69 |  |  |
| 68 | 49 |  |  |
| 69 | 40 |  |  |
| 70 | 51 |  |  |
| 71 | 79 | GO:0003735structural constituent of ribosome (18) | 0.001 |
|  |  | GO:0003723 - RNA binding (15) | 0.001 |
| 72 | 42 |  |  |
| 73 | 62 |  |  |
| 74 | 140 |  |  |
| 75 | 186 |  |  |
| 76 | 40 |  |  |
| 77 | 263 | GO:0005200 - structural constituent of cytoskeleton (8) | 0.013 |
| 78 | 89 |  |  |
| 79 | 119 |  |  |
| 80 | 41 |  |  |
| 81 | 53 |  |  |
| 82 | 183 |  |  |
| 83 | 19 |  |  |
| 84 | 63 |  |  |
| 85 | 142 |  |  |
| 86 | 101 |  |  |
| 87 | 131 |  |  |
| 88 | 26 |  |  |
| 89 | 60 |  |  |
| 90 | 117 | GO:0003735 - structural constituent of ribosome (22) | 0.001 |
|  |  | GO:0003723 - RNA binding (21) | 0.001 |
| 91 | 195 |  |  |
| 92 | 77 | GO:0006968 - cellular defense response (7) | 0.001 |
|  |  | GO:0019835 - cytolysis (4) | 0.001 |
|  |  | GO:0004872 - receptor activity (16) | 0.005 |
| 93 | 45 |  |  |
| 94 | 17 |  |  |
| 95 | 99 | GO:0009058 - biosynthetic process (24) | 0.001 |
|  |  | GO:0003735 - structural constituent of ribosome (19) | 0.001 |
|  |  | GO:0003723 - RNA binding (16) | 0.001 |
|  |  | GO:0006119 - oxidative phosphorylation (6) | 0.001 |
|  |  | GO:0015078 - hydrogen ion transporter activity (6) | 0.006 |
| 96 | 23 |  |  |
| 97 | 29 |  |  |
| 98 | 165 |  |  |
| 99 | 17 |  |  |
| **Bicluster** | **Probesets** | **GO ID - term (recovered genes)** | **Corrected p-value** |
| 100 | 40 |  |  |
| 101 | 119 |  |  |
| 102 | 126 |  |  |
| 103 | 64 |  |  |
| 104 | 45 |  |  |
| 105 | 144 |  |  |
| 106 | 12 |  |  |
| 107 | 107 |  |  |
| 108 | 59 |  |  |
| 109 | 59 | GO:0042113 - B cell activation (5) | 0.037 |
| 110 | 91 |  |  |
| 111 | 61 | GO:0003735 - structural constituent of ribosome (7) | 0.008 |
| 112 | 158 |  |  |
| 113 | 79 |  |  |
| 114 | 27 |  |  |
| 115 | 59 |  |  |
| 116 | 80 |  |  |
| 117 | 77 |  |  |
| 118 | 35 |  |  |
| 119 | 133 |  |  |
| 120 | 47 |  |  |
| 121 | 115 |  |  |

The TANGO algorithm (Tool for Analysis of GO enrichment) was used to identify the biological significance of 121 biclusters from 9,856 selected probe sets (see material and merthods for details). Processes with corrected p value < 0.05 were considered significant [36].
